# Reprogramming Short-Chain Fatty Acid Metabolism Mitigates Tissue Damage for Streptococcus pyogenes Necrotizing Skin Infection

**DOI:** 10.21203/rs.3.rs-3689163/v1

**Published:** 2023-12-23

**Authors:** Michael Caparon, Wei Xu, Tara Bradstreet, Zongsen Zou, Suzanne Hickerson, Yuan Zhou, Hongwu He, Brian Edelson

**Affiliations:** Washington University School of Medicine; Washington University School of Medicine; Washington University School of Medicine; Washington University School of Medicine; Washington University School of Medicine; Jiangxi Normal University; Central China Normal University; Washington University School of Medicine

**Keywords:** Streptococcus, macrophage, neutrophils, short-chain fatty-acid, IL-10, skin-infection, fermentation, HDAC, therapeutic

## Abstract

Disease Tolerance (DT) is a host response to infection that limits collateral damage to host tissues while having a neutral effect on pathogen fitness. Previously, we found that the pathogenic lactic acid bacterium *Streptococcus pyogenes* manipulates DT using its aerobic mixed-acid fermentation (ARMAF) pathway via the enzyme pyruvate dehydrogenase (PDH) to alter expression of the immunosuppressive cytokine IL-10. However, the microbe-derived molecules that mediate communication with the host’s DT pathways remain elusive. Here, we show that ARMAF inhibits accumulation of IL-10-producing inflammatory cells including neutrophils and macrophages, leading to delayed bacterial clearance and wound healing. Expression of IL-10 is inhibited through streptococcal production of the short chain fermentation end-products acetate and formate, via manipulation of host acetyl-CoA metabolism, altering non-histone regulatory lysine acetylation. A bacterial-specific PDH inhibitor reduced tissue damage during murine infection, suggesting that reprogramming carbon flow provides a novel therapeutic strategy to mitigate tissue damage during infection.

## INTRODUCTION

Resistance to infection requires immune functions that reduce pathogen burden. Equally crucial within the realm of immunity is a function that remains neutral with respect to pathogen fitness, known as “Disease Tolerance” (DT). This aspect of immunity is often coupled to induction of cellular and tissue regenerative processes and functions to balance tissue immunopathology against the anti-pathogen response [1]. Overall, the severity of an infectious disease is influenced by the balance between the effector and DT arms of the immune system [2, 3]. It is well-established that bacterial pathogens can actively manipulate the host’s anti-pathogen response [4]. Whether they can also manipulate DT to promote fitness is less well-understood.

Previously, we found that the pathogenic lactic acid bacterium *Streptococcus pyogenes* (group A streptococcus) can manipulate DT in patterns that reflect how carbon is distributed among its several pathways of central carbon metabolism [5]. This Gram-positive bacterium is responsible for over 2.5 million cases of pharyngitis (“strep throat”) and 20,000 invasive infections annually in the USA and over 500,000 deaths world-wide, despite its sensitivity to beta-lactam antibiotics [6]. In addition, it causes several post-infection sequela including rheumatic fever, rheumatic heart disease and acute glomerulonephritis [7]. Its ability to cause this wide range of pathologies relies on its ability to exploit a diverse array of host niches [6] using the metabolic plasticity of its central carbon metabolism [8]. As a lactic acid bacterium, *S. pyogenes* exclusively relies on fermentative metabolism to reduce pyruvate for regeneration of NAD^+^ consumed by glycolysis. Its canonical pathway uses homolactic fermentation and the enzyme lactate dehydrogenase (LDH) to reduce pyruvate into lactate. However, *S. pyogenes* can also reduce pyruvate via two parallel arms of mixed-acid fermentation. The first functions only under anaerobic conditions and involves the enzyme pyruvate-formate lyase (PFL) and the second functions only under aerobic conditions using the pyruvate dehydrogenase enzyme complex (PDH). Carbon flux through these pathways dictates the composition of the short-chain fatty end-products of fermentation, including lactate, acetate and formate, which each have immunoregulatory properties [9, 10], and can influence *S. pyogenes* virulence factor expression through metabolic-sensing transcription factors such as Mga and CcpA [11].

For example, virulence factor regulation involving CcpA is tied to its central role in regulating carbon-catabolite repression, the process through which bacteria fine-tune carbon flux to make the most efficient use of available carbon source substrates [12, 13]. CcpA functions as a transcriptional repressor of metabolic and virulence genes and mutants de-repressed by deletion of CcpA had a diminished ability to grow in tissue, leading to less damage and accelerated bacterial clearance [13]. Mutants expressing a CcpA variant engineered to constitutively repress gene expression also had an reduced ability to damage tissue [13]. However, these mutants were not attenuated for their ability to grow in tissue [13], showing that growth and tissue damage are not always directly proportional and implying that *S. pyogenes* carbon flux regulates the host’s DT response [13].

To further dissect this relationship, we used a murine model of soft tissue infection to examine single and pair-wise mutants that constrained carbon flux through all combinations of the three major *S. pyogenes* fermentation pathways. While the canonical lactic acid pathway (via LDH) had a minimal contribution to virulence, the anaerobic mixed-acid fermentation (via PFL) was essential for growth in tissue. In contrast, its aerobic mixed-acid fermentation (ARMAF) had no impact on bacterial growth, but did alter the severity of tissue damage through altering the balance between the proinflammatory cytokine TNFα and the immunosuppressive cytokine IL-10 [5]. These data support a model in which central carbon flux, influenced by the availability of oxygen and specific carbon sources, is used by *S. pyogenesto* regulate its disease-causing abilities. The recent development of synthetic PDH inhibitors with selectivity demonstrated through molecular docking, mutagenesis, enzymatic assays, and inhibition kinetic analysis [14–17] suggests that it will be possible to therapeutically-manipulate bacterial carbon flux during infection.

In the present study, we examined the mechanisms by which ARMAF alters DT, including how the cellular innate immune response is reshaped by PDH to alter IL-10 expression, how PDH changes the production of short-chain fatty acid end-products to alter IL-10 expression, and how these fermentation end-products act to regulate DT via altering non-histone regulatory lysine acetylation. Finally, we show how reprogramming central carbon flux using a prokaryotic-specific PDH inhibitor can mitigate tissue damage during a necrotizing skin infection. These data help to explain how environmental and nutritional factors in different host tissues can influence the severity of disease and provide a new therapeutic strategy to mitigate tissue damage by regulation of DT during invasive *S. pyogenes* infection.

## RESULTS

### PDH is required to promote ulcer progression and latestage infection.

**Figure 1:**
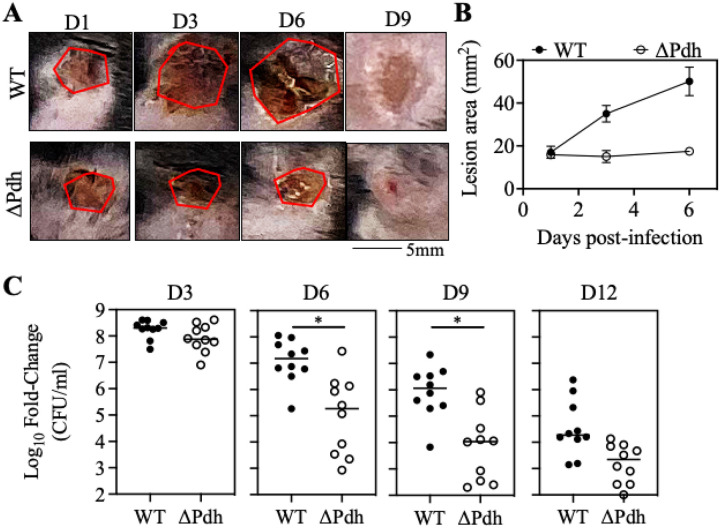
PDH is required to promote ulcer progression and late-stage infection. C57BL/6J mice were subcutaneously infected with 10^7^ CFU WT or ΔPdh and monitored daily over 12 days for tissue damage (**A–B**) and bacterial burden (**C**). (**A**) Ulcer lesion areas from representative images are outlined by a red line. (**B**) Lesion size at the indicated time points was determined by measuring the area of draining ulcer. Mean and SEM is shown (n=10 per group). (**C**) Bacterial burden in tissue was measured as the number of recoverable CFUs. Each symbol represents an individual mouse, pooled from two independent experiments. *, *P* < 0.05.

Using an established murine model of subcutaneous ulcer infection that allows an independent assessment of tissue damage (ulcer area) vs. bacterial burden (colony forming units [CFUs]), we previously reported that by 3 days post-infection (dpi), ulcers caused by wide type (WT) streptococci were significantly larger than those caused by the DPdh mutant, despite the latter yielding a bacterial burden that was not significantly different from WT ([5], see Fig. 1A, B). To examine the influence of ARMAF on late-stage infection and resolution, we compared WT to DPdh bacteria through 12 days of infection. By 1-dpi for both WT and DPdh, an initial region of induration at the site of inoculation ulcerated and developed an eschar. WT lesions expanded continuously to reach a peak on day 6 (Fig. 1A, B). In contrast, the progression of DPdh lesions stalled and did not increase over their 1-dpi size (Fig. 1A, B). Around 9-dpi, eschars began to slough off, revealing newly generated tissue and by 12-dpi hair had re-grown to complete the healing process. Clearance of bacterial burdens initiated at 4–5-dpi. However, despite obtaining the same peak burden to WT at 3-dpi, DPdh was cleared at a faster rate with significantly lower burdens apparent by 6-dpi, which continued over the 12-day period (Fig. 1C). The distinct growth/damage profiles of WT vs. DPdh at both the late and early stages of infection suggests that PDH is affecting DT possibly through manipulation of the immune response.

### ARMAF alters composition of the cellular immune response.

**Figure 2:**
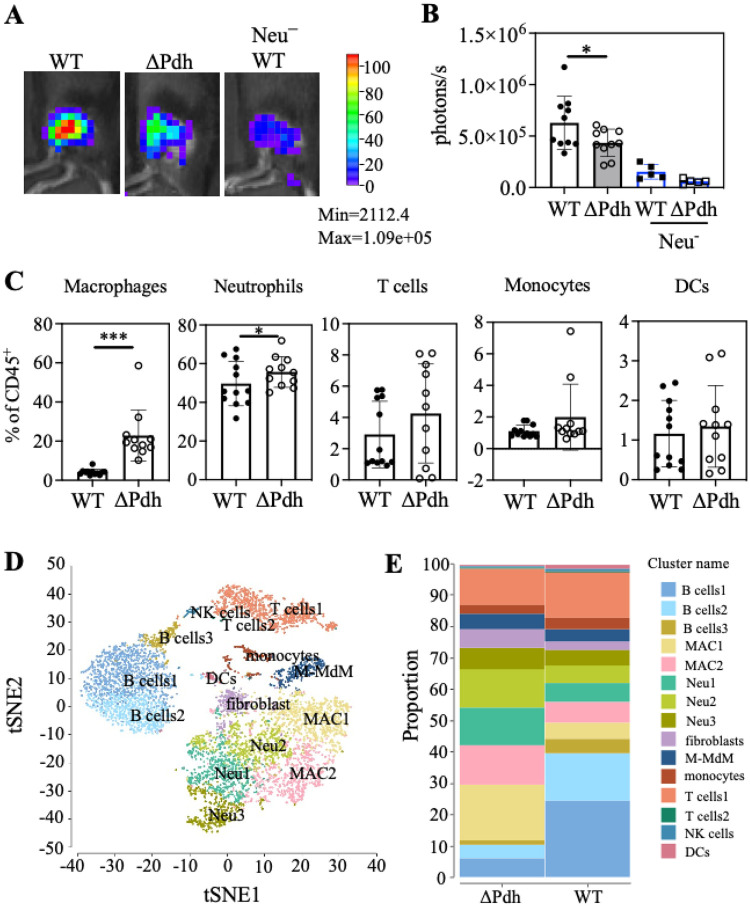
Lack of PDH induces low levels of ROS and causes a macrophage/neutrophil-dominated immune response. (**A**) C57BL/6 mice were subcutaneously infected with 10^7^ CFU WT or ΔPdh and luminol bioluminescence of ROS measured 1h post infection. Shown are bioluminescence images from representative ulcers. (**B**) Collective bioluminescence data. (**C**) The distribution of specific cells types in the total CD45+ population of cells isolated from the ulcer with minimal surrounding tissue at 3-dpi was evaluated by flow cytometry, as shown. DCs, dendritic cells. Each symbol represents an individual mouse, pooled from two independent experiments. *, *P* < 0.05; ***, *P*<0.001. (**D**) tSNE clustering analysis of all cells pooled from WT and ΔPdh from a single-cell RNA sequencing (scRNA-seq) analysis of immune cell populations in the ulcer at 3-dpi. Different cell sub-identities are indicated by color and labeled. (**E**) Relative proportion of cells with different sub-identities observed during infection by WT or ΔPdh identified by scRNA-seq. Abbreviations: MAC, macrophages; Neu, neutrophils; M-MdM, monocyte-derived macrophages; NK, natural killer cells; DCs, dendritic cells.

To test this hypothesis, we compared WT and DPdh strains during the very initial stages of infection using luminol-bioluminescence imaging [18, 19] to track reactive oxygen species (ROS)-producing cells at the site of infection. At 1-hour post-infection (hpi), significantly more ROS-producing cells were elicited by WT as compared to DPdh (Fig. 2A, B). Depletion of neutrophils using an anti-Gr1 antibody significantly reduced luminol bioluminescence for both WT and mutant strains, indicating that this early response was predominantly due to neutrophils (Fig. 2A, B). To examine other immune populations, single cell suspensions from the lesions and surrounding tissue were analyzed by flow cytometry (for gating strategies, see **Fig. S1**). By 3-dpi, the number of CD11 b^+^F4/80^+^ macrophages was 5-fold higher in DPdh-infected vs. WT-infected skin (Fig. 2C). At this time-point neutrophils were the predominant CD45^+^ cell type and were 1.5-fold higher in DPdh- vs. WT-infected cutaneous tissue. There were no significant differences in the population sizes of other myeloid or lymphoid cell types analyzed, including T cells, monocytes, and dendritic cells (Fig. 2C). Single-cell RNA sequencing of one million cells from 3-dpi WT and DPdh-infected skins identified 15 distinct cell clusters which were assigned to designated cell types by their canonical gene markers (**Fig. S2**). By collapsing cell clusters based on t-distributed stochastic neighbor embedding (t-SNE) we obtained 9 subcategories of immune cells (Fig. 2D). All immune cells were found in both WT- and ΔPdh-infected skins samples; however, ΔPdh-infection results in 2-fold higher levels of macrophages and neutrophils (Fig. 2E). Taken together, these data suggest that ARMAF affects the numbers of macrophages and neutrophils present at the site of infection.

### ARMAF is required to avoid immune surveillance.

**Figure 3:**
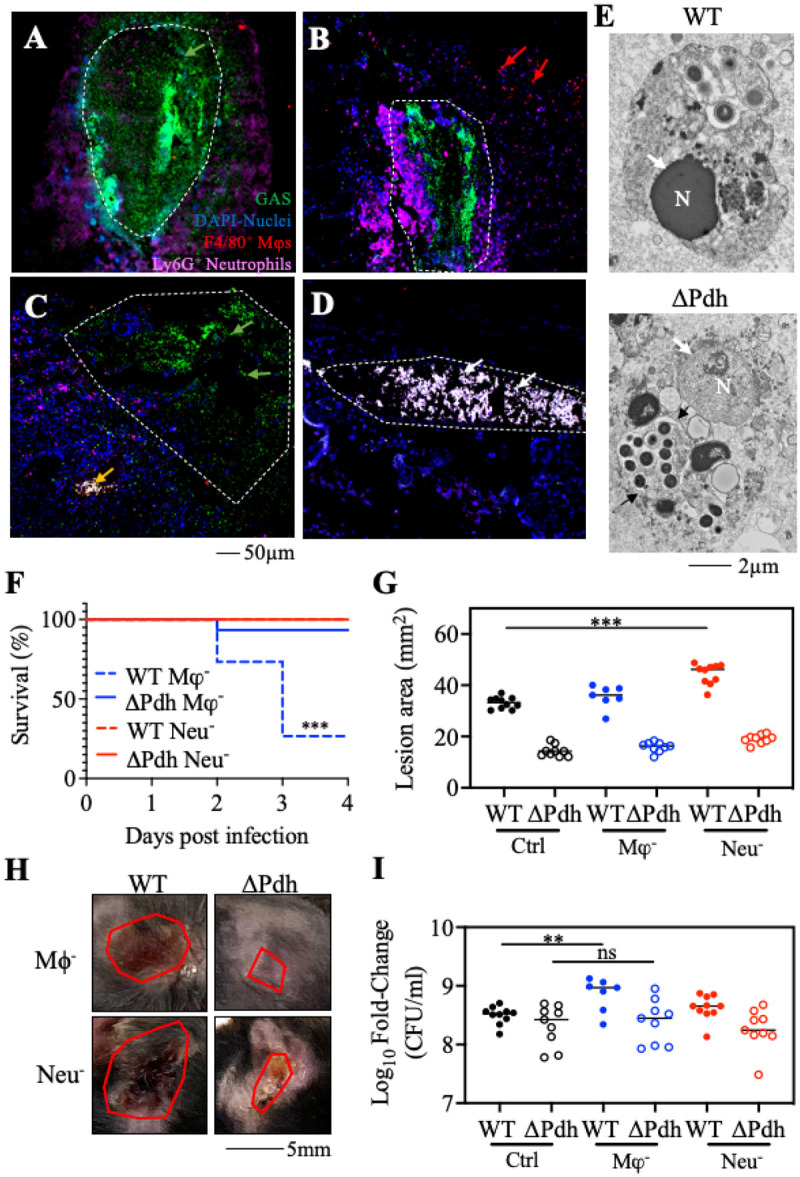
PDH contributes to extracellular growth and immune cell-dependent bacterial clearance. Formalin-fixed tissue sections from C57BL/6J mice infected with (**A, C**) WT or **(B, D**) ΔPdh were prepared following (**A, B**) 1-dpi or (**C, D**) 3-dpi. Sections were examined by fluorescence microscopy following staining with anti-F4/80 PE, anti-Ly6G Alexa Fluor 647, anti-Group A Streptococcus FITC and DAPI. Shown are extracellular bacteria (green arrows), bacteria-free macrophages (red arrows), macrophages with intracellular bacteria (yellow arrows) and merged image showing macrophages and neutrophils with intracellular bacteria (white arrows). (**E**) Transmission electron micrographs of representative macrophages infected by WT and ΔPdh as indicated. N, nucleus; Black arrow, phagosomal membrane. (**F**) Survival rate, (**G, H**) tissue damage and (**I**) bacterial burden for C57BL/6J mice following depletion of macrophages (Mφ^−^) or neutrophils (Neu^−^) and subsequently subcutaneously infected with bacteria. Data are pooled from two individual experiments and each symbol represents an individual mouse. *, *P* < 0.05; **, *P*<0.01; ***, *P*<0.001; ns: not significant.

Immunofluorescence was then used to directly observe interactions between streptococcal bacteria and recruited immune cells in infected tissue. Consistent with the data presented above, neutrophils were the predominant cell in WT lesions at 1-dpi, which surrounded a central core of the lesion where streptococcal bacteria were growing in an extra-cellular niche. The central core itself consisted mostly of bacteria and contained few intact inflammatory cells (bacteria appear green, neutrophils appear purple, Figs. 3A, S3). While neutrophils also surrounded DPdh bacteria in a central region, considerably more macrophages were observed in the surrounding tissue (red arrows, Fig. 3B). At 3-dpi, WT streptococci continued to be predominantly extra-cellular and partially breached into deeper tissues (Fig. 3C). In contrast, while the DPdh mutant had a similar tissue burden as WT at this time point (see Fig. 1A) and were constrained to a defined lesion, the streptococci were mostly located intracellularly in macrophages and/or neutrophils (Fig. 3D), as indicated by the white appearance of the merged macrophage, neutrophil, and bacterial fluorescent channels (see **Fig. S3**). Consistent with our prior study that WT and DPdh streptococci could persist intracellularly in macrophages, examination of tissue sections using transmission electron microscopy (TEM) revealed macrophages containing bacteria in membrane-bound compartments, with DPdh-infected macrophages typically containing more bacteria per cell (black arrow) with fewer signs of cell death, including nucleoli with a translucent (DPdh) vs opaque (WT) appearance (indicated by the white arrows, Fig. 3E). To test whether the influx of macrophages or neutrophils contributed to host resistance and DT, mice were treated to deplete macrophages (with clodronate liposomes) or neutrophils (with anti-Gr1 antibody) at 18 hrs prior to bacterial challenge. The efficacy of depletion was confirmed by assessing cell percentages in the spleens of all mice using flow cytometry (**Fig. S4**). Mice depleted of macrophages were highly susceptible to infection with WT *S. pyogenes,* showing significantly reduced survival by 3-dpi (70% mortality) as compared to DPdh-infected mice (10% mortality), while mice depleted of neutrophils all survived infection with both strains of bacteria (Fig. 3F). Analysis of DT revealed that when compared to the absence of treatment, depletion of neutrophils exacerbated tissue damage by WT bacteria (Fig. 3G, H), while depletion of macrophages exacerbated bacterial burdens by WT bacteria (Fig. 3I). However, DPdh mutant-induced tissue damage and burdens did not increase when either neutrophils or macrophages were depleted (Fig. 3G–I). Thus, while both WT and DPdh strains exhibited similar tissue burdens by 3-dpi, these data revealed distinct roles for macrophages and neutrophils in WT strain infections. Furthermore, macrophages and neutrophils played distinct roles in WT vs. DPdh mutant bacterial infections.

### ARMAF blocks anti-inflammatory and wound-healing responses.

**Figure 4:**
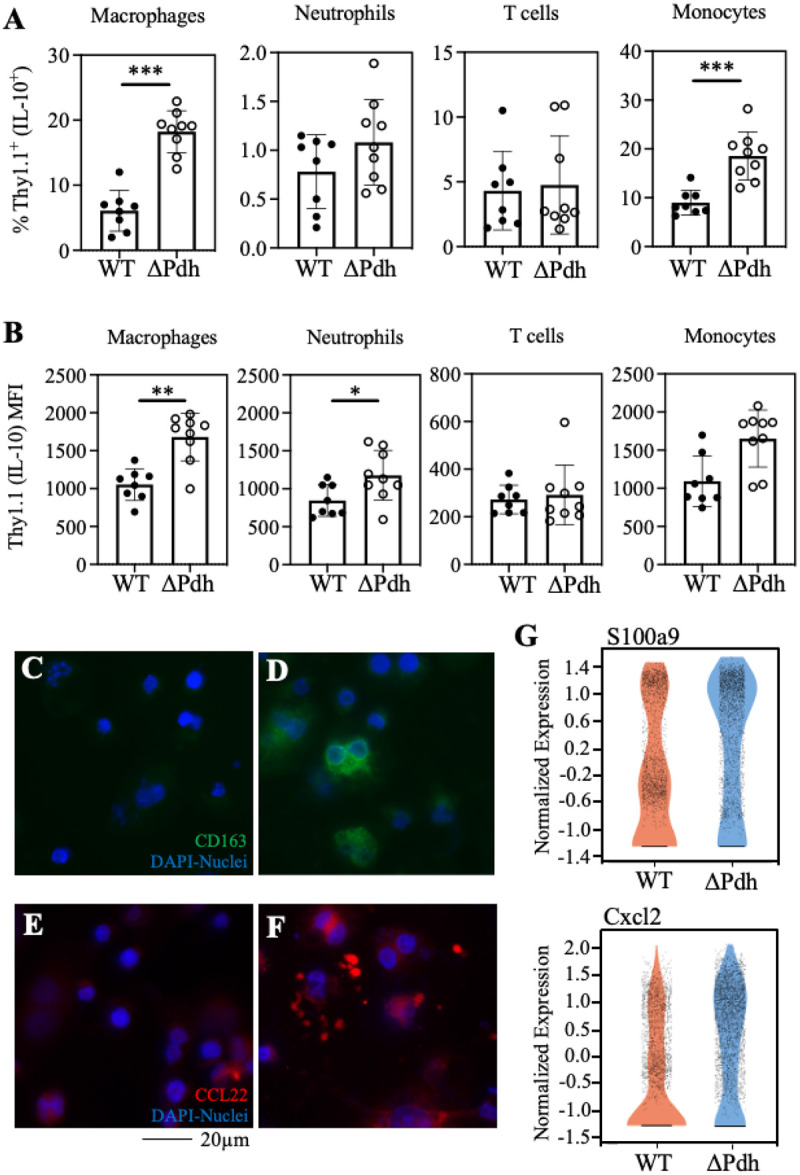
PDH blocks the anti-inflammatory and wound-healing responses to infection. 10 BiT^+^ IL10-reporter mice were used to monitor expression of IL-10 in various immune cell populations during subcutaneous infection. Quantitation of Thy1.1 by FACS is a proxy for expression of IL-10. (**A**) For each immune cell type listed, the percentage of the total number of cells expressing Thy1.1 at 3-dpi is shown. (**B**) Levels of Thy1.1 expression at 3-dpi in the indicated cell populations as determined by mean fluorescent intensity (MFI). The geometric mean ± standard deviation is shown. (**C, D**) Immuno-fluorescent microscopy was used to detect CD163 (FITC) and (**E, F**) CCL22 (Alexa Fluor 594). All samples were stained with DAPI to visualize nuclei. (**G**) Violin plots of normalized transcripts per cell for *S100a9* and *Cxcl2 from* all cells analyzed in the scRNA-seq dataset. Each dot represents a single cell. For the data shown in panels **A** and **B**: *, *P* < 0.05; **, *P*<0.01; ***, *P*<0.001.

ARMAF via PDH is required for *S. pyogenesto* selectively repress production of the anti-inflammatory cytokine IL-10 without altering expression of the pro-inflammatory cytokine TNFα [5]. To identify the cell types responsible for increased IL-10 expression, we used Tg IL-10 bacterial artificial chromosome (BAC)-in transgene (10BiT) IL-10 reporter mice, where IL-10-expressing cells display Thy1.1 on their surface [20]. Using Thy1.1 expression as a proxy for IL-10 expression, we observed both a higher frequency of Thy1.1 ^+^ macrophages, monocytes, and neutrophils in ΔPdh- vs. WT-infected skin (Fig. 4A) and that these macrophages and neutrophils expressed more Thy1.1 on a per-cell basis (Fig. 4B). In contrast, Thy1.1 expression and numbers of Thy1.1-expressing T cells were similar between WT- and DPdh-infected cutaneous tissue (Fig. 4A, B). In macrophages, expression of IL-10 is associated with the M2-like phenotype that promotes anti-inflammatory responses [21]. Analysis of RAW 264.7 macrophages infected *in vitro* revealed enhanced expression of the M2-associated markers CD163 and CCL22 [22] in DPdh-infected compared to WT-infected cells (Fig. 4C–F, S5). Examination of the single cell RNA sequencing dataset for markers associated with the wound-healing response [23] revealed higher expression of several reparatory signaling genes during DPdh infection, including *S100a9*and *Cxcl2* in myeloid cells (Fig. 4G). Together, these data show: 1. Myeloid, not lymphoid cells, are the source of increased IL-10 in DPdh infection, 2. That carbon flux through PDH is required to depress anti-inflammatory and wound-healing responses.

### SCFA’s produced by ARMAF alter levels of host acetyl-CoA, affecting lysine acetylation to regulate IL-10 expression.

**Figure 5:**
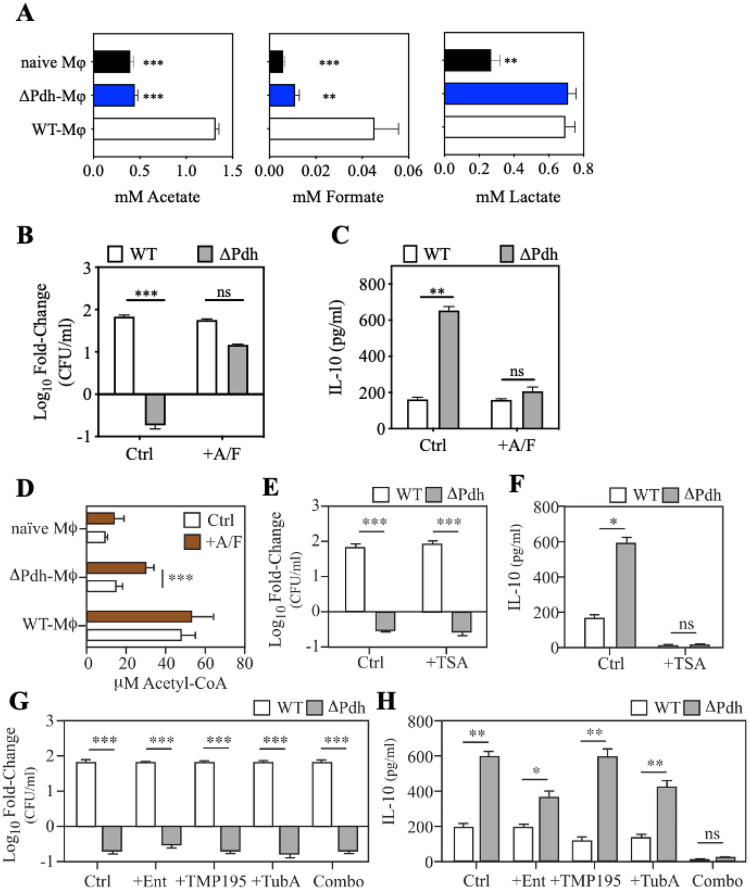
IL-10 expression is influenced by acetyl-CoA levels and the activity of histone deacetylases (HDACs). (**A**) The concentration of 3 major SCFAs (acetate, formate, lactate) in the supernatant of RAW 264.7 macrophages infected with WT, ΔPdh or uninfected (naive Mφ), as determined following 4 hrs of infection. (**B**) Cultured RAW 264.7 macrophages were infected by the indicated strains in absence (Ctrl) or presence (+A/F) of acetate (1 mM) and formate (50μM) and the net change in CFUs recovered between time 0 and 4 hrs of infection determined, as shown. (**C**) From the same infected RAW 264.7 cultures, the concentration of IL-10 in cell supernatants was determined, as shown. (**D**) Levels of intracellular acetyl-CoA in RAW 264.7 macrophages infected with WT, ΔPdh or uninfected (naïve Mφ) in the absence (Ctrl) or presence (+A/F) of acetate (1 mM) and formate (50μM) following 4 hrs of infection. (**E**) Net change in CFUs recovered between time 0 and 4 hrs of infection in the presence of the pan HDAC inhibitor TSA or (**G**)inhibitors specific for the various HDAC classes as described in the text. (**F**)Concentration of IL-10 in supernatants from infected RAW 264.7 cells in the presence of TSA or (**H**) various specific HDAC inhibitors. Inhibitors are as follows: +TSA (Trichostatin A, 5μM), +Ent (Entinostat, 50μM), +TMP195 (20μM), +Tub (Tubastatin, 50μM), Combo (Entinostat + TMP195 + Tubastatin A). Data presented represents the mean and standard error of the mean derived from three independent experiments. *, *P* < 0.05; **, *P*<0.01; ***, *P*<0.001; ns: not significant.

Constraining carbon flux through mutation of PDH alters the composition of the short-chain fatty acid (SCFA) end-products of fermentation, most notably a significant reduction in levels of acetate and formate [5]. Similar trends of low acetate and formate levels were found in the supernatant of infected macrophages (Fig. 5A). Since SCFAs have immunoregulatory properties [9], the effect of these SCFAs on DT was evaluated by complementing DPdh infection of RAW 264.7 macrophages *in vitro* by addition of exogenous acetate and formate. Supplementation restored a WT phenotype to DPdh infection, with WT levels of both persistence and inhibition of IL-10 expression (Fig. 5B–C). While the effect of formate on host cells is not well-understood, acetate can affect macrophage gene expression by a number of sensing pathways, including Toll-like receptors, G protein-coupled receptors, and adenosine receptors [24–26]. However, several antagonists targeting these pathways had no effect on IL-10 expression in response to DPdh infection (**Fig. S6–S7**). Alternatively, acetate can be utilized by host cells by conversion to acetyl-CoA, which then serves as a co-factor for a wide-range of cellular functions including gene regulation [27]. To determine whether acetyl-CoA contributes to regulation of IL-10 expression, acetyl-CoA levels were examined in infected RAW 264.7 macrophages, which revealed that macrophages infected by DPdh mutant had significantly lower levels of acetyl-CoA as compared to WT infection (Fig. 5D). Supplementation with acetate and formate significantly enhanced acetyl-CoA levels for infection by DPdh, but not for WT bacteria (Fig. 5D). Acetyl-CoA acts to regulate gene expression as a co-factor for acetylation of regulatory lysine residues on a broad range of proteins, including histones, transcription factors, chaperones, and other regulatory factors [28]. Regulation is influenced both by levels of acetyl-CoA and the activity of histone deacetylases (HDACs), which remove acetyl groups on a broad range of regulatory proteins in addition to histones [29]. To determine if acetylation contributes to regulation of IL-10 expression, infected RAW 264.7 macrophages were treated with Trichostatin A (TSA), a broad-spectrum inhibitor of zinc-dependent HDACs [30]. While having no significant effect on WT or DPdh persistence in RAW 264.7 macrophages (Fig. 5E), TSA treatment inhibited IL-10 expression to below the level of detection by infection of either WT or DPdh (Fig. 5F). There are 18 HDAC enzymes, which are organized into 5 distinct classes of which classes I, IIa, IIb, and IV are zinc-dependent [31]. Treatment with inhibitors selective for several zinc-dependent classes, including Entinostat (class I; HDACs 1, 2, 3, 8), TMP195 (class IIa; HDACs 4, 5, 7, 9) and Tubastatin A (class IIb; HDAC 6), did not affect bacterial persistence alone or when combined (Fig. 5G) and did not significantly affect IL-10 expression on an individual basis (Fig. 5H). However, the combination of all three inhibitors phenocopied TSA treatment, inhibiting IL-10 expression to near-background levels (Fig. 5H). Taken together, these data indicate that: 1. *S. pyogenes* manipulates disease tolerance using SCFAs to influence regulatory lysine acetylation by altering host acetyl-CoA metabolism; 2. Elevated levels of lysine acetylation correlate with inhibition of IL- 10 expression; and 3. The observation that suppression of IL-10 expression requires the combined action of inhibitors from several distinct HDAC classes implies that diverse regulatory pathways act redundantly to regulate IL-10.

### Reprogramming carbon flow using the PDH inhibitor H8 attenuates tissue damage.

**Figure 6:**
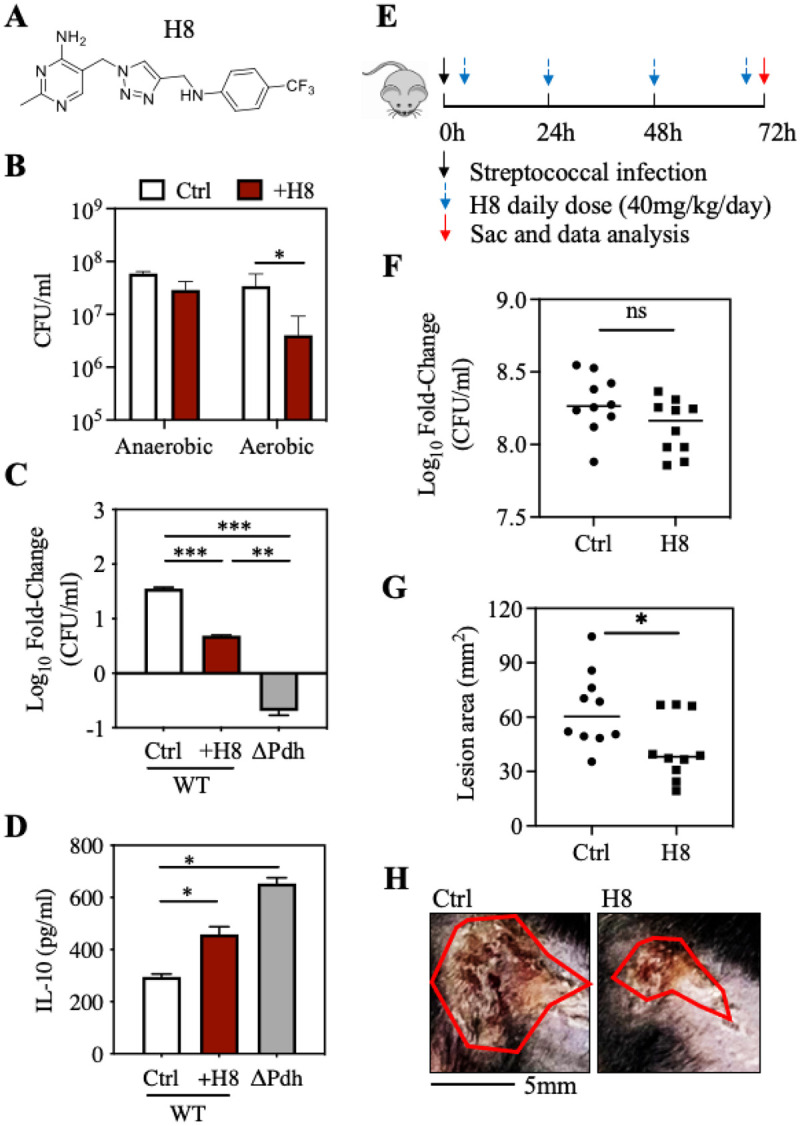
Small molecule H8 mitigates tissue damage during *S. pyogenes* infection. (**A**) Structure of H8. (**B**) *S. pyogenes* WT strain were grown in ThyB with or without 20 mg/ml H8, CFU were determined after 18 hrs of incubation under aerobic or anaerobic conditions. (**C**) Cultured RAW 264.7 macrophages were infected by WT or ΔPdh in absence (Ctrl) or presence (+H8) of 20 mg/ml H8 with net change in CFUs between time 0 and 4 hrs of infection and (**D**) concentration of IL-10 in the supernatants determined. (**E**) C57BL/6J mice were subcutaneously infected with 10^7^ CFU WT, and H8 were injected daily into the site of infection, as shown for determination of (**F**) Bacterial burden at 3-dpi and (**G**) Lesion size determined by measuring the area of draining ulcer. (**H**) Images from representative ulcers outlined with a with red line. *, *P* < 0.05; **, *P*<0.01; ***, *P*<0.001; ns: not significant

To test the hypothesis that therapeutic inhibition of PDH can attenuate tissue damage during *S. pyogenes* infection of soft tissue, we examined a library of 20 compounds with high inhibitory activity against bacterial, but not human, PDH [14, 16, 17]. Since PDH is required for aerobic growth [5], the various compounds’ ability to inhibit *S. pyogenes PDH* was assessed under aerobic growth conditions, identifying compound H8 (2-methyl-5-((4-(((4-(trifluoromethyl)phenyl)amino)methyl)-1H-1,2,3-triazol-1-yl)methyl)pyrimidin-4-amine) (Fig. 6A) as most effective in inhibition of aerobic growth (Fig. 6B). H8 effectively inhibited WT growth during macrophage infection (Fig. 6C) and elicited significantly higher levels of IL-10 in WT-infected macrophages vs. untreated macrophages (Fig. 6D). To test H8 *in vivo,* mice challenged subcutaneously with WT *S. pyogenes* (0 hr) immediately received a subcutaneous injection of H8 at the site of infection, followed by daily injections of H8 at the same site (Fig. 6E). When analyzed at 3-dpi, H8-treated mice had a similar burden of infection as compared to untreated (vehicle treated) mice (Fig. 6F). However, lesions areas of H8-treated mice were significantly reduced relative to untreated mice (Fig. 6G) with less tissue damage (Fig. 6H).

## DISCUSSION

In this study, we have determined the mechanism through which a pathogenic bacterium can manipulate the host’s DT response in patterns influenced by oxygen and carbon flow through its central carbon metabolism. We show how a small molecule inhibitor of a fermentative enzyme can reprogram carbon flow to exploit DT control mechanisms to mitigate tissue damage during soft tissue infection. Further studies into how pathogens can actively manipulate DT responses may provide novel approaches for therapeutic management of tissue destructive infections.

Comparison of WT to DPdh *S. pyogenes* in a murine model of infection supports the following model for how central carbon metabolism manipulates DT. For WT, streptococcal bacteria are predominantly extracellular. However, a minor population of bacteria is phagocytosed by monocyte-derived macrophages recruited to the site of infection. Using ARMAF, bacteria persist in these cells to produce high levels of acetate and formate, which are metabolized by macrophages [32, 33], resulting in elevated levels of acetyl-CoA. High levels of acetyl-CoA stimulate acetylation of lysine residues on regulatory proteins involved in IL-10 regulation with the result that IL-10 expression is repressed. Consequently, the ratio of IL-10 relative to the pro-inflammatory cytokine TNFa is low, delaying the wound healing response to promote greater damage to tissue. In the absence of PDH activity, either through mutation or from chemical inhibition, only low levels of acetate and formate are produced by the streptococcal bacteria, macrophage acetyl-CoA levels are not elevated, and lysine acetylation of IL-10 regulatory protein(s) is reduced. As a result, when the IL-10/TNFα ratio is elevated, it leads to the recruitment of a higher number of macrophages and neutrophils that produce IL-10 at the infection site. This, in turn, triggers a wound-healing program aimed at halting the advancement of tissue damage and expediting the kinetics of wound healing. This model that links the host DT response to bacterial metabolic carbon flow can also help to explain how *S. pyogenes* infections can dramatically differ in severity depending on the site of infection in response to host-derived cues, as tissue-specific differences in the availability of carbon sources and oxygen can influence carbon flow and thus, how the bacterium manipulates host DT responses.

Due to its broad-spectrum anti-inflammatory activity and its expression by a variety of immune cells, regulation of IL-10 expression plays a key role in balancing DT against the pro-inflammatory anti-pathogen response [2]. However, the factors involved are poorly understood. It is known that regulation is multi-factorial and includes numerous transcription factors and post-transcriptional mechanisms (reviewed [34]). An example of the former is Bhlhe40, which acts to repress IL-10 expression during *Mycobacterium tuberculosis* infection [35]. Here we show that HDAC activity plays an important role in regulation of IL-10 expression. In the best characterized mechanism of HDAC regulation of gene expression, histone acetylation leads to an unwinding of chromatin structure to stimulate transcription (reviewed [28]). HDAC inhibitors elevate levels of histone acetylation resulting in enhanced transcription [28]. However, HDAC inhibitors had the opposite effect during *S. pyogenes* infection, indicating that elevated levels of lysine acetylation acts to suppress, rather than stimulate, IL-10 expression. This suggests that the PDH-dependent regulatory mechanism does not involve acetylation of histones. Consistent with this, we found that infection with WT or DPdh did not result in elevated acetylation of histone H3, nor did supplementation with acetate + formate. Although as expected, higher levels of acetylation were observed upon treatment with the HDAC inhibitor TSA (**Fig. S8**). Consistent with this, it has been observed that in macrophages, histone phosphorylation rather than acetylation plays an important role in control of IL-10 gene expression [36]. Since HDACs also have multiple non-histone protein targets, including transcription factors, signal transduction mediators, DNA repair enzymes, nuclear import regulators, chaperone proteins and other inflammation mediators [29], it is likely that *S. pyogenes* is targeting a non-histone regulator. Furthermore, since a combination of inhibitors specific for several different classes of zinc-dependent HDAC families was required to phenocopy the broad-spectrum inhibitor TSA, it is likely that HDACs downstream of multiple regulatory pathways act redundantly in IL-10 regulation.

By providing a mechanism for how *S. pyogenes* can actively manipulate the DT response and how this affects the cellular characteristics of the innate immune response, the data presented here extend studies showing that cooperation between macrophages and neutrophils is required for innate defense against *S. pyogenes* during infection of soft tissue. It has been shown that resident macrophages effectively phagocytose and kill *S. pyogenes* in the absence of opsonic antibodies, while monocytes migrate to the site of infection and differentiate into macrophages assisting with bacterial clearance [37–39]. Monocyte-derived macrophages play an important role in regulating the neutrophilic response, which acts to compartmentalize the infection to prevent systemic spread [37–39]. Macrophages also must balance the expression of pro- and anti-inflammatory cytokines to regulate the wound healing response [22]. How macrophages achieve this balance during infection is unknown; however, our analysis suggests that disruption of bacterial aerobic metabolism can significantly interfere with this balance. Consistent with our prior report [5], the data suggest that while *S. pyogenes* is predominantly an extracellular pathogen in soft tissue, that DT is regulated by a minor population of bacteria that are internalized by monocyte-derived macrophages. These intracellular bacteria manipulate macrophage immunoregulatory activities to regulate macrophage polarization and macrophage-neutrophil cooperation such that ΔPdh-infected tissue contains twice as many macrophages as WT-infected tissue, which also become infected by intracellular bacteria, leading to higher expression of IL-10 and an accelerated wound-healing response.

Studies of the microbiome space have shown that microbial SCFAs play an important role in shaping health and disease through immunomodulatory effects [40]. For example, acetate can enhance innate immune responses through signaling via the membrane-associated free fatty acid receptor 2 [41]. Formate can manipulate patterns of methylation to regulate mitophagy and inhibit the polarization of macrophages to pro-inflammatory phenotypes [42]. Lactate produced by *Staphylococcus aureus* biofilm can inhibit HDAC11 to reprogram host immunity to promote persistent infection [43, 44]. In this study, we show a novel mechanism through which SCFAs regulate immunity by manipulation of host cell levels of acetyl-CoA, which impacts regulatory lysine acetylation. Unlike the nuclear processes targeted by other SCFA regulatory pathways, *S. pyogenes* SCFA production may be affecting non-nuclear pathways, as neither infection, nor supplementation with acetate and formate, alters histone acetylation (**Fig. S8**). This is consistent with studies indicating that there is compartmentalization of production of acetyl-CoA in the mitochondria and cytoplasm vs. the nucleus [27, 45–47]. Identification of the pathway(s) targeted by streptococcal SCFA metabolism will be important for understanding how a pathogen can manipulate the DT response.

Due to its role in regulation of DT, PDH becomes an attractive target for therapeutic interventions in severe invasive diseases. In addition to *S. pyogenes,* PDH is critical for the growth of numerous pathogenic bacteria [48, 49]. PDH is an enzyme complex that converts pyruvate into acetyl-CoA, which consists of three enzymes [5]. Of these, component E1a catalyzes the initial irreversible reaction of the complex and is highly conserved between eukaryotic and prokaryotic organisms. However, using the structure of the *Escherichia coli* PDH E1a enzyme, a combination of molecular docking, site-specific mutagenesis and enzymatic assays has resulted in the synthesis of a series of compounds with highly selective binding to the prokaryotic enzyme that has significant anti-bacterial activity against several Gram-negative species [14, 16, 17]. We have shown that *S. pyogenes* growth in soft tissue is primarily supported by mixed-acid fermentation through PFL [5], so the failure of chemical inhibition to reduce CFUs during soft-tissue infection was not unexpected. However, carbon flux through the PDH arm of mixed acid fermentation does influence DT to regulate levels of tissue damage [5]. Here we show that a selective PDH inhibitor was effective in reprogramming carbon flux to alter patterns of tissue damage. Further refinement to increase the efficacy of targeting the Gram-positive PDH E1a enzyme may result in effective therapies for tissue destructive infections where rapid tissue damage limits perfusion of conventional antibiotics to the site of infection and retards the healing process, resulting in unwanted surgical interventions [50–53]. Therapies that reduce tissue destruction in combination with standard-of-care antibiotics could improve treatment outcome.

## MATERIALS and METHODS

### Ethics.

All animal experiments were approved by the Institutional Animal Care and Use Committee (protocol #16–1119 and #22–0307). Procedures were performed according to all institutional policies, Animal Welfare Act, NIH guidelines and American Veterinary Medical Association guidelines on euthanasia. Adult (6–8-week-old) female C57BL/6J and derivative mice, were maintained at Washington University School of Medicine animal facilities. 10BiT IL-10 reporter mice were maintained in a specific pathogen-free facility [20].

### Bacterial strains.

Where indicated, *S. pyogenes* HSC5 [54] and isogenic mutant DPdh [5] were cultured in Todd Hewitt +1 % Yeast Extract (THY) broth [55] or C medium supplemented with 0.2% glucose [55]. Quantitation of *S. pyogenes* CFUs utilized THY medium solidified by the addition of 1.4% agar [55], which was incubated at 37°C anaerobically using a commercial atmospheric container (GasPak^™^ EZ, catalog #BD 260001). All cultures were seeded from overnight cultures in C medium to an initial OD_600_ = 0.05, which were then incubated for the various times indicated in the text.

### Murine subcutaneous ulcer model of infection.

Infection of 6–8-week-old female C57BL/6 and 10BiT^+^ mice followed a well-established protocol [56]. Briefly, mice received a subcutaneous injection of ~ 10^7^ CFU of the indicated bacterial strains into the thigh and the areas of the resulting ulcers determined following 72 hrs of infection from analysis of digital images using ImageJ as described [56]. Bacterial burdens in lesions were assessed by excision and homogenization of infected tissues and spot plating aliquots of the tissue homogenate [57]. Procedures of H8 treatment were shown in Fig. 6E.

### Macrophage infection.

RAW 264.7 macrophages were grown in Dulbecco’s Modified Eagles Medium (DMEM, Sigma) with 10% fetal bovine serum supplemented with antibiotics (1% Penicillin-Streptomycin) at 37°C in an atmosphere of 5% CO_2_. For infection, the bacterial inoculum was prepared as described above for murine infection, and after replacing media with antibiotic free media, bacteria were added to macrophages cultured in 12-well dishes at an MOI of 10:1 which were then immediately subjected to centrifugation at 500 × g for 1 min. and placed in an incubator for 1 hr. Wells were then washed twice with Dulbecco’s PBS, then DMEM containing antibiotics (100μg/ml Gentamicin) was added for 5 minutes and washed 3 x with PBS before the cells were incubated for an additional 2–4 hrs in antibiotic-free DMEM. For supplementation, acetate/formate (Sigma-Aldrich, #32319, 71539) or acetylation inhibitors (TSA: Sigma-Aldrich, T1952; TMP195: Selleck, #S8502; Tub A: Selleck, #S8049; Ent: Selleck, #S1053; H8 is custom-synthesized using the formula from Fig. 6A [14, 16, 17]) were added at the beginning of infection. Cells were harvested using a cell scraper and collected by centrifugation at 1,000 × g for 5 min, were resuspended in 1ml dH_2_O at pH 11 and lysed using a cup sonicator for 20 sec. Bacteria in the resulting suspension were collected by centrifugation (6,000 × g, 5 min) and resuspended in 100 μl of C-media for determination of CFUs. Net fold-change in CFU was determined by comparison to cells harvested after the addition of antibiotic-containing medium.

### Luminol Assay.

Bioluminescence images were captured using the In Vivo Imaging System (IVIS) Spectrum (Perkin Elmer, Santa Clara, CA, USA) and analyzed using IVIS imaging software (Perkin Elmer, version 2.6.1). Ten minutes before imaging, animals received an intraperitoneal (IP) injection of 200 mg/kg luminol sodium salt (Sigma-Aldrich, A4685).

### Flow Cytometry.

To prepare single cell suspensions, infected ulcer with minimal surrounding tissue were removed aseptically and placed in ice-cold PBS. Samples were mechanically dissociated using a GentleMACS^™^ dissociator in C Tubes (Miltenyi Biotec, 130–096-334) according to manufacturer’s protocol [58]. The resulting cell suspension was filtered through a 40μm cell strainer (Corning Falcon, 352340). For staining, all antibodies were used at a dilution of 1:200. Single-cell suspensions were preincubated with anti-CD16/CD32 Fc Block antibody (BioXCell, BE0307) in PBS for 10 min at RT before staining with the following antibodies. Antibodies were obtained from BioLegend and included: FITC anti-CD45.2 (109806), BV605 anti-MHC II (107639), APC anti-CD64 (139306), PB anti-Ly6C (128014), and BV510 anti-Thy1.1 (202535). The following anti–mouse antibodies were obtained from Tonbo Biosciences: PE anti-F4/80 (50–4801-U100), PercpCy5.5 anti-Ly6G (65–1276-U025), APC-Cy7 anti-CD11c (25–0114-U025), PE-Cy7 anti-CD11b (60–0112-U100). Alexa Fluor^™^ 700 anti-CD3ɛ was from Invitrogen (56–0033-82). Cells were stained for 20 min at 4°C, washed, and fixed in 4% paraformaldehyde (Electron Microscopy Sciences) in PBS for 20 min at 4°C. Cell counts were determined by hemocytometer [35]. Flow cytometry data were acquired on an LSR Fortessa cytometer (BD) and analyzed using FlowJo software (TreeStar, version 10.8.1). Gating strategies are depicted in **Fig. S1**.

### Transmission Electron Microscopy.

For ultrastructural analyses, samples were fixed in 2% paraformaldehyde/2.5% glutaraldehyde (Ted Pella Inc, Redding, CA) in 100 mM sodium cacodylate buffer for 2hr at room temperature and then overnight at 4°C. Samples were washed in sodium cacodylate buffer and postfixed in 1% osmium tetroxide (Ted Pella Inc.) for 1hr at room temperature. After three washes in dH_2_O, samples were stained en bloc in 1% aqueous uranyl acetate (Electron Microscopy Sciences, Hatfield, PA) for 1 hr. Samples were then rinsed in dH_2_0, dehydrated in a graded series of ethanol, and embedded in Eponate 12 resin (Ted Pella Inc). Ultrathin sections were cut to a thickness of 95nm with a Leica Ultracut UCT ultramicrotome (Leica Microsystems Inc., Bannockburn, IL), stained with uranyl acetate and lead citrate, and viewed on a JEOL 1200 EX transmission electron microscope (JEOL USA Inc., Peabody, MA) equipped with an AMT 8-megapixel digital camera and AMT Image Capture Engine V602 software (Advanced Microscopy Techniques, Woburn, MA).

### Macrophage or Neutrophil depletion.

To deplete macrophages, mice were intraperitoneally injected with 1mg liposomal clodronate or control liposomes (Fisher Scientific, NC1488571) [59]. To deplete neutrophils, mice were intraperitoneally injected with 500 μg monoclonal anti-Ly6G (BioXCell, BE0075–1) or anti-Gr1 (BioXCell, BE0075) antibody, or rat IgG2a isotype control (BioXCell, BE0089) diluted in sterile PBS 18 hrs before infection [60]. Depletion efficiency was assessed by FACS as described in the text and Fig. S4.

### Western Blot Analyses.

Following 4hr of infection, RAW 264.7 macrophages were collected by gentle scrape in parallel with uninfected RAW 264.7 cells. Total cell lysates were subjected to a Western blot analysis using anti-H3K9 (Cell Signaling Technology #9649T) or anti-H3 (Cell Signaling Technology #4499T), and a goat anti-rabbit IgG conjugated with HRP Blots were developed and imaged using a ChemiDoc PM system (Bio-Rad), as previously described [61].

### Cytokine Quantification.

At the 4 hrs time point, supernatants from infected and uninfected RAW 264.7 cells were harvested by gentle scrape and centrifuge at 1000 × g for 5 mins, and stored at −80°C. The following day, the concentration of IL-10 and TNFα in supernatants were determined by ELISA according to the manufacturer’s protocol (R&D Systems, cat.#DY417, #DY410). Concentrations were determined by comparison to a standard curve generated using purified proteins provided in the kit.

### Measurement of SCFAs and Acetyl-CoA.

Following 4hrs of infection, RAW 264.7 cells were harvested by gentle scrape, subjected to centrifugation (1000 × g, 5 min) and the resulting supernatant fluids and cell pellets stored separately at −80°C. The concentrations of acetate (Sigma-Aldrich, MAK086), lactate (Sigma-Aldrich, MAK064), and formate (Sigma-Aldrich, MAK059) were then determined in cell supernatants. The concentration of acetyl-CoA from cell pellets was measured by a colorimetric assay using commercial kits (Sigma-Aldrich, MAK039) following the manufacturer’s protocol. SCFA and acetyl-CoA levels were compared to uninfected RAW 264.7 cells.

### Fluorescent Microscopy.

Infected skin ulcers were fixed in 10% buffered formalin (Thermo Fisher Scientific) for 24 hrs and in 70% ethanol overnight at 4°C. Samples were then paraffin embedded and sectioned by the Anatomic and Molecular Pathology Core Labs at Washington University. Immunofluorescent staining was performed as described previously [62]. PE anti-F4/80 (Tonbo, 50–4801-U100), FITC anti-Streptococcus Group A (Invitrogen, PA1–73056), Alexa Fluor^™^ 647 anti-Ly6G (BioLegend, 127609), CD163 (Invitrogen, #16646–1-AP), MDC (Invitrogen, #57226) antibodies were used. Fluorescent images were acquired using a Zeiss Axio Imager 2 microscope (Jena, Germany) equipped with a digital camera. Fluorescent intensity was measured with ImageJ software (https://imagej.nih.gov/ij/ ).

### Single cell RNA-seq.

DNA was prepared after the Gel Beads in Emulsion (GEM) generation and barcoding, followed by the GEM-RT reaction and bead cleanup steps. Purified cDNA was amplified for 11–16 cycles before being size-selected using SPRIselect beads (Beckman Coulter, B23319). Samples were then run on a Bioanalyzer (Agilent, #2100) to determine the cDNA concentration. Gene Expression libraries were prepared as recommended by the 10x Genomics Chromium Single Cell 5’ Reagent Kits User Guide (v2 Chemistry Dual Index), with appropriate modifications to the PCR cycles based on the calculated cDNA concentration. For sample preparation on the 10x Genomics platform, the Chromium Next GEM Single Cell 5’ Kit v2, 16 rxns (PN-1000263), Chromium Next GEM Chip K Single Cell Kit, 48 rxns (PN-1000286), Dual Index Kit TT Set A, 96 rxns (PN-1000215) were used. The concentration of each library was determined through qPCR utilizing the KAPA library Quantification Kit according to the manufacturer’s protocol (KAPA Biosystems/Roche) to produce cluster counts appropriate for the Illumina NovaSeq6000 instrument. Normalized libraries were sequenced on a NovaSeq6000 S4 Flow Cell using the XP workflow and a 151×10×10×151 sequencing recipe according to the manufacturer’s protocol. A median sequencing depth of 50,000 reads/cell was targeted for each Gene Expression library. Transcript alignment, counting, and inter-library normalization were performed using the Cell Ranger pipeline (10x Genomics, default settings, Version 2.1.1, GRCh38 reference). Data were analyzed using the Biomage online tool (https://www.biomage.net/).

### Statistical analyses.

Data are derived from at least 3 independent experiments, with values presented representing the mean ± SEM for each group. The difference between experimental groups were tested for significance using a two-tailed Mann-Whitney U test available in GraphPad Prism software where *, ** and *** indicates *p* < 0.05, < 0.01 and < 0.001, respectively. For all tests, the null hypothesis was rejected for *p* > 0.05.

## Data Availability

Raw data files for ScRNASeq are deposited on NCBI with BioProject ID PRJNA917702.
